# The Cognitive Information Effect of Televised News

**DOI:** 10.3389/fpsyg.2017.01165

**Published:** 2017-07-10

**Authors:** George Lăzăroiu, Aurel Pera, Ramona O. Ştefănescu-Mihăilă, Sofia Bratu, Nela Mircică

**Affiliations:** ^1^Department of Social-Human Sciences, Spiru Haret UniversityBucharest, Romania; ^2^Department of Teacher Training, University of CraiovaCraiova, Romania; ^3^Department of Economic Sciences, Spiru Haret UniversityBucharest, Romania

**Keywords:** televised news, viewer, cognitive abilities, meaning, information processing

## Abstract

The purpose of this review is to summarize the key findings which prove that the biased perceptions of viewers may provide an inaccurate image of the informational validity of televised news. The news may generate distorted recollections of what occurred in particular reported events if displayed routines influence viewers not to pay attention to the essential features of a narrative. Elaborating on Fiske and Hartley (2010), Zelizer (2010), and Gunter (2015), we indicate that the character of the news setting has altered and individuals’ news consumption routines have changed in adapting to media advancements. The news may be undergone at various psychological stages by news publics. Televised news may transmit information undeviatingly to publics that may (not) be committed successfully to memory. Our paper shows that individuals’ skills to handle information that is displayed in a linguistic configuration are influenced by their abilities in the utilization of certain symbol systems that are employed to represent notions and meanings. Televised news may shape what individuals grasp, influence their perceptions, convictions, and views regarding prevailing events and matters, and transmit knowledge and interpretation. If news stories can be jotted down in a linguistic style that sidesteps making needless processing demands and captivate news users by facilitating them to make connections with former knowledge, they may be more worthy of note and more edifying. We conclude that news narratives present a cognitive demanding task to individuals, displaying novel information regarding evolving events in a multifarious format. Broadcast news exhibits intricate contents, displaying configurations that employ excessively the cognitive abilities for information processing of viewers.

## Introduction

The biased perceptions of viewers may provide an inaccurate image of the informational validity of televised news as nearly all individuals are short of a wide-ranging knowledge of issues that have been broadcast in the news. The latter may generate distorted recollections of what occurred in particular reported events if displayed routines (Nica et al., 2016; Popescu and Predescu, 2016; Weede, 2016) influence viewers not to pay attention to the essential features of a narrative. Pre-existing issue knowledge is a significant component in connection with the intensity of novel reminiscences (Androniceanu and Drăgulănescu, 2012; Laudan et al., 2016; Mihăilă, 2016; Baldassarre et al., 2017) that are shaped out of broadcast news practices. Images may put forth an influential dominance over the viewer’s mind and if they go pear-shaped in backing the spoken information provided by television announcers, news may be rendered an insubstantial mixture or too demanding to assimilate accurately. Numerous of the implemented alterations to news narrative and display formats may undermine their information value. Some deficiencies of information from broadcast news should be attributed to viewers and their shortage of wider appropriate knowledge that may improve their proficiency in handling intricate unfamiliar information supplied to them by television announcers. As television news may have a type of cognitive effect, individuals should watch it. A lot of the broadcast information is unsuccessful in influencing viewers or disappears from their memories swiftly (Gunter, 2015).

## Is Televised News A Persuasive Communicator of Information?

The manner in which narratives are jotted down may distort the fashion news users construe the events and matters being broadcast. Publics may differ in the pre-existing cognitive abilities they own and the latter may influence their information processing tendencies. News broadcasts may establish the publics’ news agenda and define the present relevance of particular narratives, influencing the manner audiences assimilate particular events and matters (Nica et al., 2016; Popescu and Predescu, 2016; Weede, 2016) by formulating them so that to highlight particular narrative elements over others. The character of the news setting has altered and individuals’ news consumption routines have changed in adapting to media advancements. The television news may be undergone at various psychological stages by news publics, shaping what individuals grasp and influencing their perceptions, convictions, and views regarding prevailing events and matters (Lindberg, 2016; Fox, 2017; Schaefer and Northoff, 2017), and transmitting knowledge and interpretation. They also may generate events, matters, and featured individuals to be recollected displaying them more reachable in mind should they afterward be activated to consider them again. Media reporting of particular events and matters may impact their importance in the broader public consciousness. Televised news bulletins may generate misperception in the approaches of viewers via displayed routines that further the amalgamation of factual features from distinct narratives (Gunter, 2015).

News images are instrumental for publics striving to decipher problematic events of an intricate and puzzling character. Numerous broadcasters are confused regarding the role of images and how to determine which image may be pertinent or significant for a news narrative, and diverge on the usefulness of illustrative representation. Thus they perceive images not as creations but as reflectors of the issues that they reproduce (Fujiwara and Lawton, 2016; Terry, 2016; Wexler, 2017), the ascendancy of images increasing when the news grows in proportion or relevance. The strength to get used to visual images has constantly varied by news medium. News is chiefly sound information substitute that employs words as its principal means of expression and inevitably conceives pictures as harming, distorting, or counterbalancing journalism’s dependence on truthful reason. Nearly all broadcasters show partiality toward an image’s implication, counting on graphic realism to improve their reporting of the factual world (Hayes and Jeffries, 2016; Popescu et al., 2016; Xia et al., 2017) and exhibiting images in substantial amounts and importance whenever they are required to claim ascendancy for their reporting. Operating as channels of both information and memory, news images generate public interest irrespective of how thoroughly they represent what viewers may know and grasp. News images may perform via an attribute of reason (a mixture of likelihood, creativity, and responsiveness) that comes to a decision not at the image’s initial moment of exhibition but in time by various individuals putting it to distinct utilizations in novel settings. An image’s signification builds upon underlying shared dispositions available in assisting individuals comprehend what they see (Zelizer, 2010).

The realm of television is unambiguously distinct from the factual social world, but also somehow recognizably associated with it: television does not depict the apparent substantiality of the society but replicates emblematically the arrangement of values and links (Androniceanu and Drăgulănescu, 2012; Laudan et al., 2016; Mihăilă, 2016; Baldassarre et al., 2017) beneath the façade. The evidently driven character of the television sign should not direct viewers to the correspondingly essential function of usage in disclosing its meaning. Television signs may function synchronously metaphorically and metonymically, but each mode carries out a distinct role. In the first position of signification, the iconic or denotative role of the sign is metaphoric, entailing the shift from concreteness to representation. In the second position of signification, in underlying matter, the metaphoric mode is likely to prevail. Broadcast television is a main instrument by which a culture may interact with its shared self. Television is reliant upon more wide-ranging cultural mechanisms for its implications, approaches, and interpretations: it is transient, discontinuous, explicit, accurate, and intense in mode, and its messages are achieved by disparities and by the association of apparently irreconcilable symbols and its argumentation is narrated and graphic. The television medium displays an incessant flow of images that are thoroughly recognizable in configuration and form (Hellman and Majamäki, 2016; Rogers, 2016; Zapparoli et al., 2017), employs codes which are carefully associated with those by which individuals observe reality, seems to be the expected manner of perceiving the world, and exhibits the viewers’ shared selves. Television is a social construct, and its performance is the outcome of human preference, cultural judgments, and social constraints. A grasp of the manner in which television organizes and displays its depiction of reality may be adequate toward assisting individuals in comprehending the manner in which the society functions: the television discourse exhibits daily a relentlessly retailored account of social connections and cultural assessments (Fiske and Hartley, 2010).

## The Pervasiveness of Televised News and its Relevance in the Awareness of the Public

Both stimulation generated by the news and the orientation of reaction that is caused may operate autonomously and interactively to impact viewers’ feedbacks to televised news broadcasts. If newscasters present news narratives that reverberate with long-established storytelling routines, news accounts may harmonize more effortlessly into pre-designed cognitive processing pattern (Friedman et al., 2016; Peters, 2016; Tomasi et al., 2017) employed by news users. Both story choices and the character of storytelling should supplement publics at a cognitive level and involve them at a moving one. Televised news may transmit information undeviatingly to publics that may (not) be committed successfully to memory, comprising a combination of production elements over which the audiences have almost no control. Individuals’ skills to handle information that is displayed in a linguistic configuration are influenced by their abilities in the utilization of certain symbol systems (Machan, 2016a,b; Popescu, 2016; Tulloch, 2016) that are employed to represent notions and meanings. Committing to reflection any novel information acquired from individuals’ outside setting arises via several stages and entails particular cognitive mechanisms that place requests on cognitive supplies and where the latter go beyond the processing strength that is achievable at the moment of reception, individuals might choose to concentrate on particular aspects of information while disregarding or disbelieving others (Gunter, 2015).

Viewers employ images to deal with the news, depending on their strength to portray the world more accurate, attainable, and swiftly comprehensible. The challenging co-occurrence of an unexpected and fragmentary image and complementary verbal characteristics that display it more wide-ranging than it appears takes on a distinctive form in immediate succession after the wider frictions between concepts and images as interconnected or incompatible channels (Nica et al., 2016; Popescu and Predescu, 2016; Weede, 2016) of news substitute, where augmenting verbal information may be employed more readily to appraise, build up, and construe an image or dispute and disprove what the image displays. News images are present in an abundance of possible exposures, which thrust in and around the messages of news reporting, between the current and aimed features of broadcasting, and between the latter and the greater mediated setting (broadcasting has built up throughout emerging platforms of technological substitute, and reciprocity of messages are now easier between broadcasting and the other information setting). The news may stimulate likelihood, creativity, and responsiveness (Hellman and Majamäki, 2016; Rogers, 2016; Zapparoli et al., 2017) as a manner of reacting to the world. Granted broadcasting’s prominence as a main organization of documentation and recollection, news images justify interest on their own conditions. More information supplies more strength to involve expressively, creatively, and contingently with the unpredictable occurrences of the news (Zelizer, 2010).

The image on the screen should build on the supplies of ordinary verbal language. Television’s roles are somewhat contingent on and determined by the roles carried out by speech: its visual material takes substantially the configuration of paralinguistic signs formulated fundamentally from pre-televisual linguistic codes. The intrinsic psychological state of the viewer may not be the leading driver in the dissemination of television messages that are decrypted consistent with separately assimilated and culturally produced codes and conventions (Hayes and Jeffries, 2016; Popescu et al., 2016; Xia et al., 2017) that require comparable limitations of assessment on the encoders of the reports. As a medium, television is specifically appropriate to selecting a particular iconic sign such an image and deriving it into a broader sign, i.e., providing the initial sign a novel stage of culturally constant meaning (Hurd, 2016; Nica, 2016; Androniceanu, 2017; Di Domenico and Ryan, 2017): the same visual image may signify distinct things to some extent, or communicate various types of meaning, contingent upon the medium via which the image is conveyed. Television is a distinct type of reality from the culture to which viewers belong, but the latter grasp both of them equivalently, and thus they merge with each other. Watching television and participating in ordinary life are habitual and unpremeditated undertakings which most individuals involve in without aiming an intricate investigation. The cognizance individuals bring to the television screen may be a requirement for comprehending what they observe, but it is generated in viewers by what they have come across previously. The television medium is not an impenetrable scheme, complying with its own inherent regulations and being somewhat unaffected by extrinsic circumstances. There is no individual authorial coherence for the television communicator (Fiske and Hartley, 2010).

## Cognitive, Emotional, Attitudinal, and Behavioral Results Related to News Consumption

News users set their own news fabrics that resemble cognitive devices that reinforce the manner narrative aspects are systematized in their memories. The cognitive processing of news narratives necessitates news users to retain the novel information in a provisional memory repository and to appeal to longer-run comprehension of the world to supply a setting in which the novel information may be construed. The story-like structure of a news narrative organizes an evidence of the broadcast events and sets up a fabric of meanings that news users may assimilate into their memories. There are inherent characteristics to news coverage that highlight particular narrative schemes that necessitate a distinct kind of elucidative cognitive structure (Friedman et al., 2016; Peters, 2016; Tomasi et al., 2017) concerning news publics from the fabrics they may more ordinarily utilize when processing material from storytelling accounts. If news stories can be jotted down in a linguistic style that sidesteps making needless processing demands and captivate news users by facilitating them to make connections with former knowledge, they may be more worthy of note and more edifying. The publics’ concerns may be directed by the contents of news narratives and by production characteristics. Particular format components may be used to determine viewers’ interest to certain stories and to shift them toward the performance if their attention is about to diminish. Memory for news is regulated by the information processing strength (Machan, 2016a,b; Popescu, 2016; Tulloch, 2016) of individuals composing the public. The writing style embraced by broadcasters may impact how straightforwardly news publics handle reported unbiased information. The incidence of images enhances the information processing contents and may involve with stories in various manners either to improve or hinder grasp and memory for accurate content (Gunter, 2015).

Television images may operate in incomplete connection to the issues they illustrate instead of, as words do, endeavoring to supply an adequate reporting of them, being determined by eventuality and not, as words argue, by steadiness, and their material is resourcefulness and responsiveness, not coherent analysis: the more they are examined, the more chances they provide for individuals to operate through them in attributing meaning to problematic events. Television images may convincingly demand interest, exhibition, and commitment, but what happens next relies on individuals who choose to examine them, a reporting framework that reinforces them (Carter and Yeo, 2017; Fujita et al., 2017; Kocsel et al., 2017; Libey and Fetz, 2017), and a connection with a contiguous setting that requires particular maintainable information concerning its shared life. The news may not entail a separate event documented at a moment in a place but is likely to be placed throughout time and space, and thus a news narrative’s implication is reliant on various images employed to illustrate an evolving series of events. Ongoing routes of performance are depicted in any place in the news. Experiencing the problematic events of the news can happen notwithstanding what or how much an individual grasps about them. Eventuality challenges a pervasive movement in the news in the direction of the increasingly confined readings of its public events (Lindberg, 2016; Fox, 2017; Schaefer and Northoff, 2017) and indicates a significant degree of imbalance, explanatory alteration, and peculiarity as a manner of attributing meaning to broadcasting’s problematic events (Zelizer, 2010).

Television represents viewers’ ordinary approaches into a correspondingly particular, but less conventional, language system, may concentrate expressively on massive cross-cultural publics, regulating segmentations within specific communities, and reacts to the segmentations within society similarly as the latter integrates them for nearly all practical objectives. The television message is construed distinctively as soon as it is deciphered by its audiences who adjust their position in relation to the message (Fujiwara and Lawton, 2016; Terry, 2016; Wexler, 2017) and thus alter its meaning, being eloquent as soon as the semiotic codes dovetail the cultural responsiveness provided by the viewer, whose framework has a role in influencing it. The signs in television may be components of a diversity of sub-codes or registers that imply the extent of their effectiveness to the culture. Television employs a mass-produced semiotic system to convey culturally settled and established meanings (Benedikter, 2016; Layard, 2016; Diederich et al., 2017; Nica, 2017), and capitalizes on the structures viewers consistently utilize to identify their knowledge of the world in general. Television’s modes of performance are formulated from both prevailing and secondary codes, and the friction between various spheres of society is represented not so relevantly in the denotative component of the reports as in the manner the latter are displayed. Television’s meanings are achieved via the mechanisms of spoken discourse related to visual images, and not via the frameworks of formal logic: noticeable discrepancies or breaches in logic may not be weaknesses in television discourse. The latter is not fixed and neutral organically, its mode being that of rhetoric (Fiske and Hartley, 2010).

## Conclusion

Cognitive patterns of information processing clarify that the capacity of news publics to assimilate from mediated news is influenced by their cognitive processing skills. News narratives present a cognitive demanding task to individuals, displaying novel information regarding evolving events in a multifarious format. Recurrence of components of news narratives may assist viewers in recollecting them via providing them chances to handle the information they supply. Broadcast news exhibits intricate contents, displaying configurations that employ excessively the cognitive abilities (Friedman et al., 2016; Peters, 2016; Tomasi et al., 2017) for information processing of viewers. The elaboration of television productions may be expressed in relation to a series of structural and semantic characteristics that may associate together to establish the convolution and vibrancy of a televised report. Images and words perform creditably in the television news when they supply each other with elucidative backing. Televised news reports are displayed at a pace established by their producers, sustaining a vigorous regularity that is intended to maintain viewers involved, and via customary alterations in narratives and newsgathering styles and frameworks (Lindberg, 2016; Fox, 2017; Schaefer and Northoff, 2017) to reorganize viewers’ interest to the screen. News editors may assist viewers of televised news in recollecting more of what they assimilate by considering the manners that cognitive reactions to news may be affected by the fashion it is assembled and displayed. Because the information material with which individuals are challenged is more intricate (Hurd, 2016; Nica, 2016; Androniceanu, 2017; Di Domenico and Ryan, 2017), their cognitive information processing arrangements appear under superior pressure: if information is displayed more swiftly, this overloads their cognitive strength, and if news narratives exhibit more than one route of information, they affect their cognitive processing systems tremendously. These consequences on their cognitive processing systems take place when viewers are challenged by televised news (Gunter, 2015).

## Author Contributions

All authors listed have made a substantial, direct and intellectual contribution to the work, and approved it for publication.

## Conflict of Interest Statement

The authors declare that the research was conducted in the absence of any commercial or financial relationships that could be construed as a potential conflict of interest.

The reviewer LI declared a shared affiliation, though no other collaboration, with several of the authors GL, RS-M, SB and NM to the handling Editor, who ensured that the process nevertheless met the standards of a fair and objective review.
